# The Role of Phe82 and Phe351 in Auxin-Induced Substrate Perception by TIR1 Ubiquitin Ligase: A Novel Insight from Molecular Dynamics Simulations

**DOI:** 10.1371/journal.pone.0010742

**Published:** 2010-05-20

**Authors:** Ge-Fei Hao, Guang-Fu Yang

**Affiliations:** Key Laboratory of Pesticide & Chemical Biology of Ministry of Education, College of Chemistry, Central China Normal University, Wuhan, People's Republic of China; University of Queensland, Australia

## Abstract

It is well known that Auxin plays a key role in controlling many aspects of plant growth and development. Crystal structures of Transport inhibitor response 1 (TIR1), a true receptor of auxin, were very recently determined for TIR1 alone and in complexes with auxin and different synthetic analogues and an Auxin/Indole-3-Acetic Acid (Aux/IAA) substrate peptide. However, the dynamic conformational changes of the key residues of TIR1 that take place during the auxin and substrate perception by TIR1 and the detailed mechanism of these changes are still unclear. In the present study, various computational techniques were integrated to uncover the detailed molecular mechanism of the auxin and Aux/IAA perception process; these simulations included molecular dynamics (MD) simulations on complexes and the free enzyme, the molecular mechanics Poisson Boltzmann surface area (MM-PBSA) calculations, normal mode analysis, and hydrogen bond energy (HBE) calculations. The computational simulation results provided a reasonable explanation for the structure-activity relationships of auxin and its synthetic analogues in view of energy. In addition, a more detailed model for auxin and Aux/IAA perception was also proposed, indicating that Phe82 and Phe351 played a pivotal role in Aux/IAA perception. Upon auxin binding, Phe82 underwent conformational changes to accommodate the subsequent binding of Aux/IAA. As a result, auxin enhances the TIR1-Aux/IAA interactions by acting as a “molecular glue”. Besides, Phe351 acts as a “fastener” to further improve the substrate binding. The structural and mechanistic insights obtained from the present study will provide valuable clues for the future design of promising auxin analogues.

## Introduction

**Figure 1:**
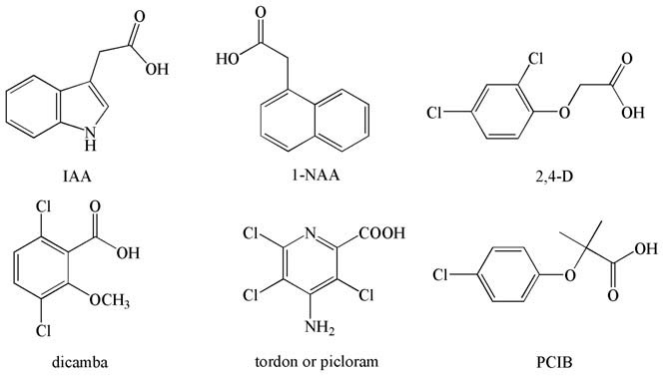
Chemical structures of IAA and some synthetic auxins.

As a pivotal plant hormone, auxin controls many aspects of plant growth and development [1]–[7] by modulating gene expression and, thus, leading to changes in cell division, expansion, and differentiation [8], [9]. Indole-3-acetic acid (IAA) is the major naturally occurring auxin. In addition, several synthetic auxins have also been developed, including 2,4-dichlorophenoxyacetic acid (2,4-D), 1-naphthalene acetic acid (1-NAA), 2-methoxy-3,6-dichlorobenzoic acid (dicamba), 4-amino-3,5,6-trichloropicolinic acid (tordon or picloram), α-(*p*-chlorophenoxy) isobutyric acid (PCIB, an antiauxin), and so on (Figure **1**) [10]. These chemically diverse molecules share two common characteristics: a planar aromatic ring and a side chain with a carboxyl group. Although auxin is known as one of the most important “signaling messengers” in the plant kingdom, the detailed action mechanism of auxin with its receptor remains one of the most interesting questions in plant biology.

Auxin binding protein 1 (ABP1) is the first protein thought to be a possible auxin receptor [11]–[16]. A potential physiological mechanism for auxin-ABP1-induced changes of the plasma membrane has recently been found by a molecular modeling study [17], but the detailed physiological role of this protein in auxin-mediated signal regulation has not been realized [12], [18]. In addition, some aspects of auxin-regulated transcription are well understood [19], [20]. For example, two families of transcription factor proteins have been identified in the response of transcription: auxin response factors (ARFs) and Auxin/Indole-3-Acetic Acid (Aux/IAA) transcriptional repressor proteins [21]–[26]. Recently, Transport inhibitor response 1 (TIR1), the F-box protein subunit of the ubiquitin-ligase complex (SCF^TIR1^), was identified as a true auxin receptor. It was also revealed that auxin binds directly to TIR1 and increases the binding between Aux/IAA and TIR1 [27], [28]. Most importantly, a series of crystallographic structures of TIR1, auxin, and Aux/IAA complex were very recently reported, which revealed that auxin, acting as ‘molecular glue’, enhanced TIR1-Aux/IAA interactions by filling a hydrophobic cavity at the interface between TIR1 and Aux/IAA [26], [29], [30]. Actually, structural analysis of TIR1 in complex with auxin and the Aux/IAA protein uncovered the pivotal role of auxin on the degradation of Aux/IAA proteins that actually activated ARF-induced DNA transcription [14], [31]–[34]. Thus, the first structural model of a plant hormone receptor had been proposed. However, the detailed mechanism of the dynamic conformational changes that key residues of TIR1 undergo during auxin and substrate perception by TIR1 is still unknown.

In the present study, various computational techniques, including molecular dynamics (MD) simulations on complexes and the free enzyme, the molecular mechanics Poisson Boltzmann surface area (MM-PBSA) calculations, normal mode analysis (NMA), and hydrogen bond energy (HBE) calculations were integrated to uncover the detailed molecular mechanism of the Aux/IAA perception process. The crystal structures of TIR1 in complex with IAA; 2,4-D; and 1-NAA were used as the initial structures for MD simulations which were carried out to investigate the stability of protein conformation, especially, the conformational flexibility of Loop-2 of TIR1 and its relation to the binding. In addition, a reasonable explanation for the structure-activity relationships of auxin and its analogues was provided by the results of MM-PBSA and NMA calculations. Based on the results of computational simulation and energy calculation, a detailed Aux/IAA perception model was proposed, which explained the key roles of co-factor inositol hexakisphosphate (InsP6) and residues Phe82 and Phe351.

## Methods

### System Preparation

The initial structures of IAA; 2,4-D; and 1-NAA-TIR1 complexes used in our computational studies came from the X-ray crystal structures (pdb entry: 2P1Q, 2P1N, and 2P1O) in the Protein Data Bank [30]. One crystallization water molecule involved a water-bridge between residues and ligands was retained for each system, and other crystallization water molecules were removed. Standard Amber ff99 force field was assigned to the protein, and the general AMBER force field (gaff) was assigned to the ligands including the co-factor InsP6 [35]–[37]. The partial atomic charges of ligands were calculated using the am1-bcc method implemented in the Antechamber module of the Amber8 package [38]. Default protonation states were set to the ionizable residues at *p*H = 7. Considering the overall electroneutrality of the system, an appropriate number of Na^+^ ions were added to the most electronegative areas around the protein. Then, each system was embedded in the truncated octahedron box of TIP3P water molecules with a 8.0 Å buffer along each dimension [39], resulting in a system with ∼88000 atoms. To avoid edge effects, periodic boundary conditions were applied in all calculations.

For each system, energy minimizations and MD simulations were performed using the Sander module of the Amber8 program. First, the complex was frozen and the solvent molecules and counterions were allowed to move during a 5000-step minimization (2000 steps of the steepest descent and 3000 steps of the conjugated gradient minimization). Then, all atoms were energy-minimized with 10000 steps (5000 steps of the steepest descent and 5000 steps of the conjugated gradient minimization). After the minimization stage, the system was slowly heated from T = 10 to 300 K in 40 ps and equilibrated in 160 ps before a sufficiently long MD simulation at room temperature. Finally we run the MD simulations of each system at 1 atm and 300 K for 2 ns or longer to make sure that we obtained a stable MD trajectory for each of the simulated structures. The time step used for the MD simulations was set to 2.0 fs and the coordinates were collected every 1 ps. In the simulation, the covalent bonds to hydrogen atoms were constrained using the SHAKE algorithm [40]. The Particle Mesh Ewald (PME) method was employed to calculate long-range electrostatic interactions [41], [42]. The cutoff distances for the long-range electrostatic and van der Waals energy terms were set at 10.0 Å.

### MM-PBSA Calculation

The calculations for the binding free energies of each system were based upon snapshots taken from a single trajectory of the complex MD simulation. A total of 100 snapshots were taken from the last 0.5 ns trajectory with an interval of 5 ps for each system. The counterions and water molecules (waters related to the crucial hydrogen bond were not included) were stripped. The MM-PBSA approach implemented in the Amber8 program was used to calculate the relative binding free energies of ligands to the TIR1 protein. The detailed description of this method can be found elsewhere [43]. Generally, the protein-ligand binding free energy was calculated using the following equations:
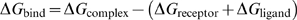
(1)where Δ*G*_complex_, Δ*G*_receptor_, and Δ*G*_ligand_ are the free energies of the complex, the receptor, and the ligand, respectively. Each can be evaluated as follows:
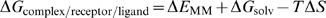
(2)Δ*G*_complex/receptor/ligand_ was evaluated as a sum of the changes in the molecular mechanical (MM) gas-phase binding energy (Δ*E*_MM_), solvation free energy (Δ*G*_solv_), and entropy term (−TΔ*S*). The molecular mechanics gas-phase binding energy (Δ*E*_MM_) can be calculated by equation 3, where Δ*E*_val_, Δ*E*_ele_ and Δ*E*_vdw_ represent the internal energy contribution from bonds, angles and torsions, electrostatic and van der Waals interactions, respectively. The solvation energy Δ*G*_solv_ can also be separated into two parts (equation 4): Δ*G*_PB_ (the electrostatic contribution to the solvation free energy) and Δ*G*_np_ (nonpolar contribution to the solvation free energy). The DelPhi program [44] with PARSE radii [45] was used to evaluate Δ*G*_PB_. The grid spacing of the cubic lattice was 0.5 Å. The dielectric constants used for the interior and exterior were 1 and 80, respectively, and 1000 iterations were performed for the linear PB equation. The nonpolar contribution to the solvation free energy can be determined using the function of the solvent accessible surface area (SASA) [45], [46], with parameters *γ* = 0.00542 kcal/Å^2^ and *β* = 0.92 kcal/mol (equation 5).
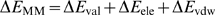
(3)

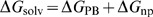
(4)

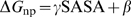
(5)


In this work, we calculated the binding free energies of each complex using the MM-PBSA method. The conformational entropies are important contributions to the receptor-ligand binding. Therefore, the NMA was performed to estimate the conformational entropy change upon ligand binding using the nmode program in Amber8 [47]. Each snapshot was fully minimized until the root-mean-square of the elements of the gradient vector was less than 1×10^−4^ kcal•mol^−1^Å^−1^. Due to the high computational cost, residues around the ligand (less than 8 Å) were used to estimate the contribution of the entropies of association and other residues were removed from each snapshot. The same strategy had been successfully applied elsewhere [48]. The calculation error bars are standard errors (SE) calculated using equation 6, where STD is standard deviation and N is the number of trajectory snapshots used in the calculation.
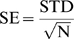
(6)


### Hydrogen bond energy calculation

Hydrogen bonds are formed according to both distance and orientation. If the distance between the donor (D) and acceptor (A) is shorter than 3.5 Å and the degree of the angle D–H…A ranges from 120° to 180°, D, H, and A with a D–H…A conformation will be considered a hydrogen bond. Generally speaking, hydrogen bonds with distance between H and A less than 2 Å are considered very strong [49]. In this work, the Ptraj module of Amber8 program was used for hydrogen bond analysis. To further understand the overall strength of the hydrogen bonding network, the HBE was calculated using the empirical HBE equation implemented in the Autodock 3.05 program [50]. The general HBE equation is
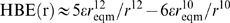
(7)where *r* is the distance between the donor hydrogen atom (H) and acceptor atom (A), *r*_eqm_ is the equilibrium internuclear separation between H and A, and *ε* is the energy well depth at *r*_eqm_. The parameters *r*_eqm_ and *ε* of the hydrogen bond acceptor were assigned by default.

## Results and Discussion

### Validation of the computational models

**Figure 2:**
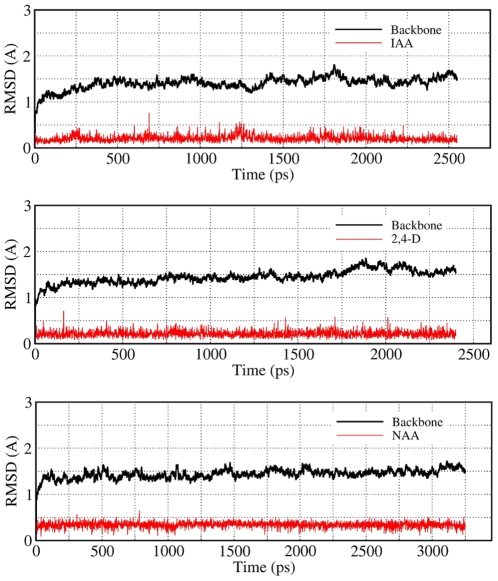
RMSD plots of the complexes during MD-simulations. RMSD of the backbone was calculated according to the coordinates of the main chain Cα atoms shown in black and the RMSD of the ligand was calculated according to the coordinates of all atoms of the ligand shown in red.

**Figure 3:**
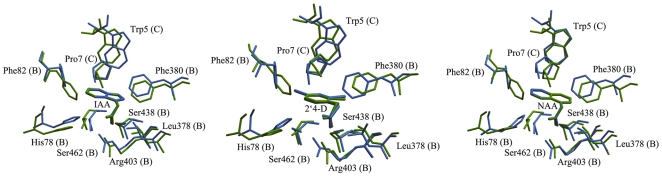
Superimposition between the MD-simulated structures and the X-ray structures. Average structures from the last 0.5 ns of the MD simulations of the complexes were superimposed on the X-ray structures via the heavy atoms of the active site. Heavy atoms of the ligands and selected neighboring residues less than 4 Å are shown in stick for the (a) IAA complex (b) 2, 4-D complex, and (c) 1-NAA complex. The crystal structure is shown in green and the MD structure is shown in blue.

To evaluate the stability of the three complexes during the MD simulation, root-mean- square deviation (RMSD) values of protein backbone atoms and the ligands related to the initial X-ray crystal structure in the whole MD trajectory were examined, as shown in Figure **2**. This clearly indicated the RMSD values of the protein backbone atoms and ligand atoms were always kept around 1.5 Å and 0.4 Å respectively, which showed that the MD-simulated binding models were stable. Therefore, to acquire an atomic view of the difference between the MD-simulated structures and crystal structures, the active site residues located within 4 Å of the ligand in the MD-simulated complex were superimposed with that of the crystal complex. As shown in Figure **3**, most of the residues in the MD-simulated complex took almost the same orientation as in the crystal complex. Only residue Phe82 in the MD-simulated complex was found to display conformational changes compared to the crystal complex, which may be attributed to the flexibility of the binding cavity. Although, other conformations may be possible for Phe82 and it cannot be excluded that the conformational change of Phe82 is responsible for the binding of Aux/IAA (the role of Phe82 will be discussed below). Most importantly, however, the conformations of the ligands in the MD-simulated complex and the crystal complex are almost the same. The RMSD values based on the heavy atoms of the active site for IAA; 2,4-D; and 1-NAA were 0.07 Å, 0.19 Å, and 0.09 Å, respectively. For the subsequent energy analysis, we saved a total of 100 snapshots from a stable MD trajectory of the last 500 ps, *i.e.* one structure every 5 ps, for each MD-simulated complex.

To further evaluate the reliability of these MD-simulated models, the binding affinity of IAA; 2,4-D; and 1-NAA with TIR1 were estimated by performing MM-PBSA calculations based on the single-trajectory MD simulations. The calculation results were compared with the experimental data, as summarized in Table **1**. The experimental data (**Δ** *G*_exp_) were estimated approximately from the reported IC_50_ values [27] by the equation **Δ** *G*≈−*RT*lnIC_50_. As shown in Table **1**, the calculated binding free energies (**Δ** *G*_cal_) were −9.84 kcal/mol, −7.65 kcal/mol, and −7.96 kcal/mol for IAA; 2,4-D; and 1-NAA, respectively. The absolute values of the calculated binding free energies not only exhibited a remarkable level of agreement with the experimental values of 9.45 kcal/mol, −8.00 kcal/mol, and −8.04 kcal/mol for IAA; 2,4-D; and 1-NAA respectively (the difference is less than 0.40 kcal/mol), they also had a consistent qualitative order (IAA>1-NAA>2,4-D) with the experimental results. These results suggested the MD-simulated models and the computational protocol tested in this study were reliable.

**Table 1:** Calculated and Experimental Energetic Data (kcal/mol) at T = 298.15 K.

	Δ*E*_ele_	bccCharge	Δ*E*_vdw_	Δ*E*_gas_	Δ*G*_solv_	ClogP	Δ*G*_PB_	−TΔS	Δ*G*_cal_	Δ*G*_exp_ a
IAA	23.92 (0.72)	−0.84	−25.28 (0.28)	−1.36 (0.68)	−23.03 (0.62)	1.08	−24.39 (0.48)	14.55 (0.53)	−9.84	−9.45
2, 4-D	50.40 (0.92)	−0.56	−29.12 (0.28)	21.29 (0.89)	−42.78 (0.85)	2.69	−21.50 (0.66)	13.85 (0.52)	−7.65	−8.00
1-NAA	54.65 (0.70)	−0.57	−28.23 (0.26)	26.42 (0.67)	−49.98 (0.62)	2.53	−23.56 (0.48)	15.60 (0.51)	−7.96	−8.04

a. The experimental values Δ*G*_exp_ were derived from the experimental IC_50_ values reported in Ref [27].

### Analysis of structure-activity relationships in view of energy

**Figure 4:**
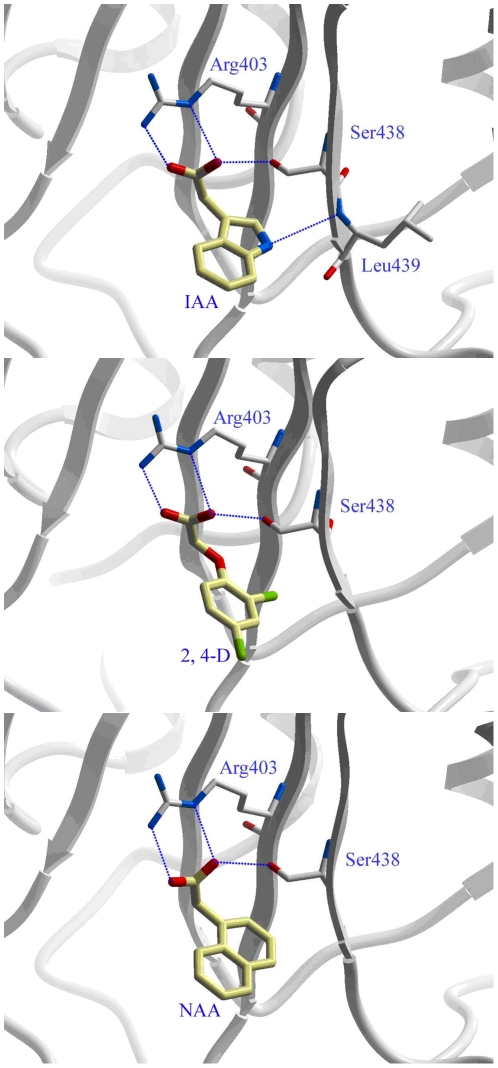
Hydrogen-bonding interaction of IAA, 2,4-D, NAA with their receptor. The H-bond distance was taken from an averaged snapshot selected from the MD simulation.

It is well known that hydrogen bonds play a pivotal role in protein-ligand interaction and make a great contribution to the total binding affinity. Therefore, we carefully analyzed the hydrogen bond networks during the whole MD simulation. As shown in Figure **4**, Arg403 and Ser438 formed several hydrogen bonds with the carboxyl group of the ligands. In addition, the nitrogen atom on the indole ring of IAA formed a hydrogen bond with the side chain of Leu439. To understand the overall strength of the hydrogen bonding network in the MD simulation, we calculated the HBE of each simulated hydrogen bond using the empirical HBE equation. As shown in Table **2**, the hydrogen bond occupancy rate and hydrogen bond distance always determine the hydrogen bond strength. Higher occupancy and shorter distance always result in lower HBE. IAA formed much stronger hydrogen bond interactions with the protein than 2,4-D and 1-NAA. Interestingly, the order of HBE (IAA>1-NAA>2,4-D) was in accordance with the strength of the binding free energies of the ligands, suggesting the hydrogen bonding interactions greatly contributed to the biological activity of auxin and its analogues.

**Table 2:** Hydrogen bond networks of the complexes.

Ligand	TIR1							
	Acceptor	Donor	%^a^	MaximumDistance	MinimumDistance	AverageDistance	HBEs^c^	THBE^d^
IAA	IAA: HN	LEU439: O	93.8	3.51	1.66	2.16	−0.47	−5.05
	IAA: O2	ARG403: HH21	100.0	2.40	1.55	1.78	−1.69	
	IAA: O3	SER438: HG	99.9	3.98	1.43	1.78	−1.79	
	IAA: O3	ARG403: HE	99.9	2.82	1.54	1.89	−1.11	
24-D	24-D: O2	ARG403: HE	99.6	2.78	1.58	1.86	−1.26	−3.25
	24-D: O1	ARG403: HH21	99.6	2.96	1.61	1.92	−1.01	
	24-D: O2	SER438: HG	95.6	4.12	1.45	2.00	−0.98	
1-NAA	NAA: O1	ARG403: HH21	100.0	2.78	1.56	1.82	−1.45	−3.73
	NAA: O2	ARG403: HE	99.8	3.09	1.60	1.94	−0.92	
	NAA: O2	SER438: HG	99.6	3.61	1.50	1.89	−1.25	
	NAA: O2	ARG403: HH21	70.8	3.78	1.76	2.57	−0.12	

^*a*^Occupancy of hydrogen bonds (The occupancy >70% were listed).

^*b*^Hydrogen bond distance (Å).

^*c*^Hydrogen bond energy (kcal/mol), calculated according to 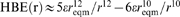, the parameters: *r*_eqm_ = 1.43 Å, *ε* = 5 kcal/mol. We calculated the HBE of every snapshot of the MD simulation and then took the average value.

^*d*^Total hydrogen bond energy (kcal/mol). The total HBE value is the average of the HBE values calculated by using the instantaneous distances in all of the snapshots.

**Figure 5:**
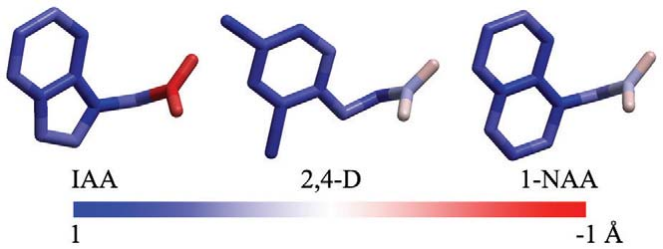
The comparison of partial charge on each atom. The color indicates the change of partial charges shown in the bottom bar.

It is very interesting to examine the interaction differences of IAA; 2,4-D; and 1-NAA with the receptor in view of energy. As shown in Table **1**, the van der Waals interaction (−25.28 kcal/mol) between IAA and the TIR1-Aux/IAA complex is a little smaller than that of 2,4-D and 1-NAA (−29.12 kcal/mol and −28.23 kcal/mol, respectively). Due to the highly electronegative charges on the InsP6, the electrostatic interactions make unfavorable contribution to ligand binding. The long range negative electric exclusion energy to IAA is 23.92 kcal/mol, which is much smaller than that of 2,4-D and 1-NAA (50.40 and 54.65 kcal/mol, respectively). Therefore, IAA should have much better binding affinity in gas than 2,4-D and 1-NAA. However, because of the low solvation free energy (−23.03 kcal/mol), IAA only has a slightly better binding affinity than 2,4-D and 1-NAA. As for the entropic change, the three systems have very similar effects with values of 14.55, 13.85, and 15.60 kcal/mol for IAA; 2,4-D; and 1-NAA, respectively. To further investigate the differences between electrostatic and solvation effects on the protein-ligand interactions, the partial charges on the carboxyl oxygen atoms and the ClogP value of each ligand were compared. As aforementioned, the carboxyl oxygen atoms of the ligands acted as acceptors to form hydrogen bonds with Arg403 and Ser438. Therefore, the partial charges on the carboxyl oxygen atoms should be very important to the strength of hydrogen bonds. As shown in Table **1** and Figure **5**, the carboxyl oxygen atoms on IAA are more negative (−0.84) than that of 2,4-D (−0.56) and 1-NAA (−0.57), which might account for IAA forming stronger hydrogen bonds with residues Arg403 and Ser438 than 2,4-D and 1-NAA. However, compared with 2,4-D and 1-NAA, IAA has a ClogP value of only 1.08 (Table **1**). As a result, IAA is more hydrophilic than 2,4-D and 1-NAA and thus has a high solvation energy. These results indicate the charges on the carboxyl oxygen atoms and the hydrophobic property of IAA should be considered in the molecular design of future auxin analogues.

### Role of Phe82 and Phe351

**Figure 6:**
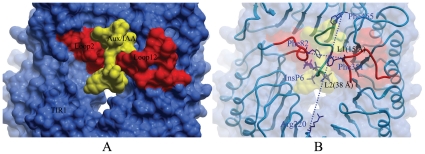
Architecture of the TIR1-LRR domain from the top view. The LRR domain is shown in blue and the two loops (loop2 and loop12) are shown in red. (A) Surface representation of the LRR domain. The substrate-binding concave groove is created by the closeness of loop2 and loop12, which nips the surface of the Aux/IAA. (B) The LRR domain are colored in blue and shown in the worm model. The distance between the Cα atoms of Phe82 in Loop 2 and Phe351 in Loop 12 was defined as transverse distance, while the distance between the Cα atoms of Arg220 and Phe465 was defined as lengthways distance.

**Figure 7:**
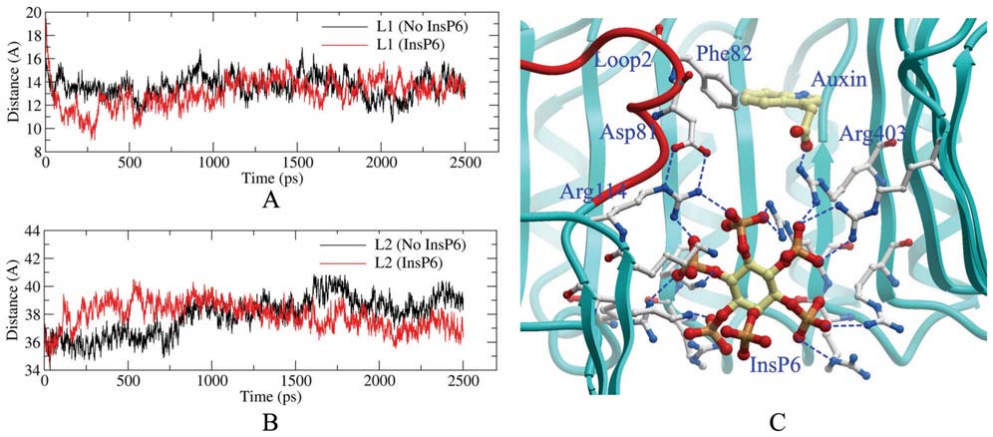
Changes of some key distances associated with InsP6 during MD simulations. The transverse distance changes (A) and the lengthways distance changes (B) of the LRR domain span along the MD simulation. The hydrogen bond networks around InsP6 (C).

From the above simulation results, we can conclude TIR1 has an important and unique characteristic: a mushroom-shaped structure with a rigid F-box motif as a ‘stem’ and a leucine-rich-repeat (LRR) domain as a ‘cap’. This mushroom shape was always kept during the whole MD simulation process. However, two loops (loop2 and loop12) (shown in Figure **6**A) in the LRR domain underwent interesting conformational changes during the MD simulation process for auxin- and substrate-binding. As shown in Figure **6**B, the changes in span distance of the LRR domain in the MD simulations of free TIR1 with or without InsP6 were analyzed to understand the conformational stabilization. As shown in Figure **7**A and 7B, significant fluctuations of the span distance can be observed in both the transverse distance (varying from 10 Å to 17 Å) and lengthwise distance (varying from 36 Å to 41 Å) of the system without InsP6, compared with the transverse (from 12 to 16 Å) and lengthwise (from 35 to 39 Å) distances of the system with InsP6. This indicates the conformation of the LRR domain is more flexible without InsP6 binding. The results from X-ray crystal structures and the MD-simulations indicated that after InsP6 binding, the conformation of the LRR domain was partly stabilized by the complicated hydrogen-bonding network between InsP6 and the receptor (shown in Figure **7**C). For example, the conserved residue Arg114 formed two hydrogen bonds with InsP6 and two hydrogen bonds with residue Asp81. So, Arg114 acted as a ‘bridge’ to link InsP6 and residue Asp81 together. The hydrogen bond interactions between Asp81 and Arg114 greatly contributed to the stabilization of Loop2. In addition, the conserved residue Arg403 formed hydrogen bonds with InsP6 and auxin. As a result, InsP6 and Auxin were linked together through the Arg403 ‘bridge’.

**Figure 8:**
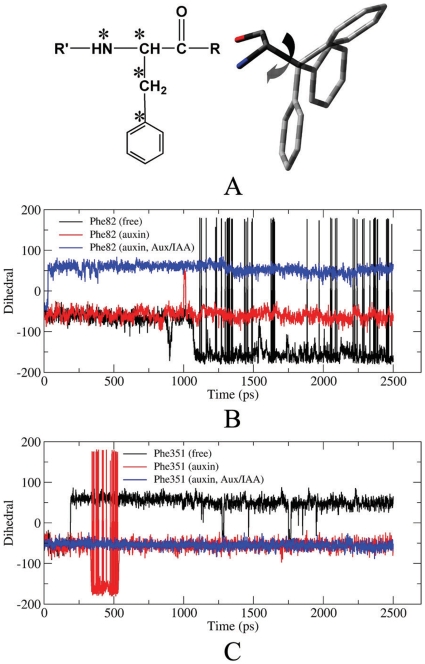
Changes of some key dihedral angles during MD simulations. Definition of dihedral angle for phenylalanine (A) and the comparison between the dihedral angle of Phe82 (B) and Phe351 (C) along the MD simulation in the free enzyme (black), enzyme with auxin (red), and enzyme with auxin and Aux/IAA (blue).

Most interestingly, our computational simulations indicated two residues, Phe82 and Phe351, underwent significant conformational changes upon the sequential binding of auxin and Aux/IAA (such as Phe82 in Figure **8**A). The side chain of Phe82 in Loop 2 had three kinds of orientations during the MD simulation for the free enzyme with two conformations occupied most of the simulation time (black line in Figure **8**B). However, upon auxin binding, the side chain of Phe82 could be induced into a favorable orientation (red line in Figure **8**B) to accommodate the subsequent binding of Aux/IAA. As a result of Aux/IAA binding, the side chain of Phe82 underwent an additional conformational change (blue line in Figure **8**B). The side chain of Phe351 was relatively unstable during the MD simulations for the free enzyme and enzyme binding with auxin (black and red line in Figure **8**C). The binding of Aux/IAA induced Phe351 to undergo a conformational change from an unstable to stable state (blue line in Figure **8**C).

### A proposed model for Aux/IAA perception

**Figure 9:**
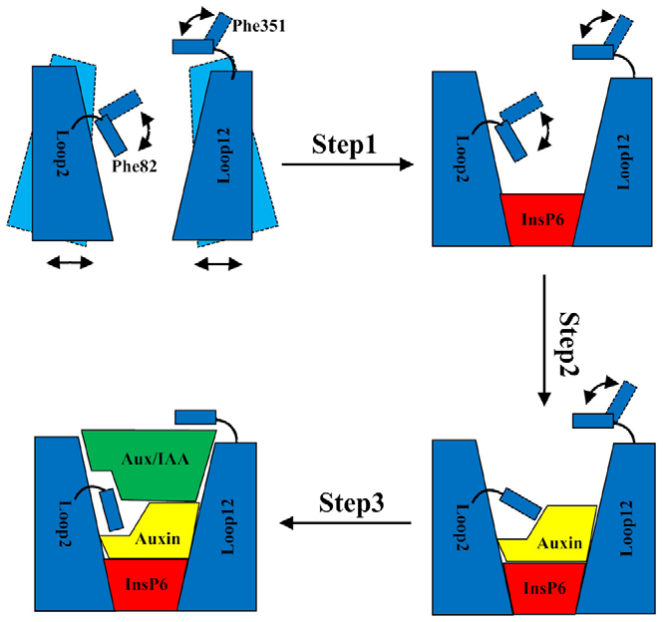
A proposed model for Aux/IAA perception.

Based on the above computational simulations, we propose a new and detailed model for Aux/IAA perception, as depicted in Figure **9**. Step 1, as a ‘conformational stabilizer’, the co-factor InsP6 binds to TIR1 to stabilize the local conformation of the LRR domain by forming hydrogen bonds with the surrounding residues. As a result of the binding of InsP6, a three-walled ‘groove’, the auxin-binding pocket, is assembled by Loop2, Loop12, and InsP6. Step 2, auxin enters this pocket and is grounded on the bottom of the ‘groove’. In addition to playing a role as a ‘molecular glue’ to increase the binding between Aux/IAA and TIR1, auxin also acts as a ‘conformation inducer’ leading Phe82 to undergo a conformational change to accommodate the subsequent binding of Aux/IAA. Step 3, Aux/IAA binds with TIR1. After the binding of Aux/IAA, Phe82 undergoes a further conformational change so that it reaches the optimum conformation for interacting with both auxin and Aux/IAA. At the same time, Phe351 acts as a ‘fastener’ to interact with Aux/IAA and prevent the substrate from leaving. Therefore, Phe82 and Phe351 play a pivotal role in Aux/IAA perception.

### Conclusion

In summary, the detailed molecular mechanism of Aux/IAA perception was uncovered by performing a series of comparative molecular dynamics simulations, MM-PBSA free energy calculations, and hydrogen bond energy calculations. According to the results from free energy and hydrogen bond calculations, the structure-activity relationships of auxin and its synthetic analogues were uncovered in view of energy. In addition, a more detailed model for Aux/IAA perception was proposed based on the results of comparative MD simulations. This model indicates Phe82 and Phe351 play a pivotal role in Aux/IAA perception. Auxin acts not only as a ‘molecular glue’ to increase binding between Aux/IAA and TIR1, but also as a ‘conformation inducer’ triggering Phe82 to undergo conformational changes to accommodate the subsequent binding of Aux/IAA. At the same time, Phe351 also acts as a ‘fastener’ to further improve substrate binding. The structural and mechanistic insights obtained from the present study will provide valuable clues for the future design of promising auxin analogues.
